# Discoidin Domain Receptor 1 Contributes to Tumorigenesis through Modulation of TGFBI Expression

**DOI:** 10.1371/journal.pone.0111515

**Published:** 2014-11-04

**Authors:** Nandini Rudra-Ganguly, Christine Lowe, Michael Mattie, Mi Sook Chang, Daulet Satpayev, Alla Verlinsky, Zili An, Liping Hu, Peng Yang, Pia Challita-Eid, David R. Stover, Daniel S. Pereira

**Affiliations:** 1 Agensys Inc., an affiliate of Astellas Pharma Inc, Santa Monica, CA, United States of America; 2 ImaginAb Inc., Inglewood, CA, United States of America; Casey Eye Institute, United States of America

## Abstract

Discoidin domain receptor 1 (DDR1) is a member of the receptor tyrosine kinase family. The receptor is activated upon binding to its ligand, collagen, and plays a crucial role in many fundamental processes such as cell differentiation, adhesion, migration and invasion. Although DDR1 is expressed in many normal tissues, upregulated expression of DDR1 in a variety of human cancers such as lung, colon and brain cancers is known to be associated with poor prognosis. Using shRNA silencing, we assessed the oncogenic potential of DDR1. DDR1 knockdown impaired tumor cell proliferation and migration *in vitro* and tumor growth *in vivo*. Microarray analysis of tumor cells demonstrated upregulation of TGFBI expression upon DDR1 knockdown, which was subsequently confirmed at the protein level. TGFBI is a TGFβ-induced extracellular matrix protein secreted by the tumor cells and is known to act either as a tumor promoter or tumor suppressor, depending on the tumor environment. Here, we show that exogenous addition of recombinant TGFBI to BXPC3 tumor cells inhibited clonogenic growth and migration, thus recapitulating the phenotypic effect observed from DDR1 silencing. BXPC3 tumor xenografts demonstrated reduced growth with DDR1 knockdown, and the same xenograft tumors exhibited an increase in TGFBI expression level. Together, these data suggest that DDR1 expression level influences tumor growth in part via modulation of TGFBI expression. The reciprocal expression of DDR1 and TGFBI may help to elucidate the contribution of DDR1 in tumorigenesis and TGFBI may also be used as a biomarker for the therapeutic development of DDR1 specific inhibitors.

## Introduction

Discoidin domain receptors (DDRs) are members of a subfamily of receptor tyrosine kinases (RTKs). This group of RTKs contains a homology domain to discoidin in their extracellular region, a lectin first described in the slime mold *Dictostelium discoideum* [1], [2], [3], [4]. The DDR1 subfamily consists of two members, DDR1 and DDR2 which are activated by triple helical collagens with distinct specificity [3], [4]; multiple isoforms of DDR1 (a–e) are generated through alternative splicing in the juxtamembrane domain [5], [6], [7], [8]. DDR1 binds to collagen via the discoidin domain and this receptor-ligand binding induces downstream signaling that regulates multiple cellular processes, which is independent of integrin [1], [8]. Oligomerization of DDR1 extracellular domain (ECD) is crucial for high affinity receptor-ligand binding [9]. Collagen-induced activation of DDR1 is unique, as it is slow and sustained over long periods of time without being degraded or inactivated by endocytosis [10]. The activation of DDR1 regulates the expression, trafficking and turnover of E-Cadherin, which participates in regulating adherent junctions [11], [12]. Yeh's group reported that DDR1 positively regulates E-cadherin mediated cell-cell junction but E-cadherin negatively regulates DDR1 activation. Since DDR1 plays a role in stabilizing cell-cell junctions through E-cadherin, it may play a valuable role in regulating collective cell migration possibly in cancer metastasis [13]. Prolonged activation of DDR1 is known to upregulate MMPs [1], [5], [14] and MMPs are associated with the invasive and metastatic potential of tumor cells by their ability to degrade extracellular matrix (ECM) [15]. Collagen-induced DDR1 activation can also promote cell survival via anti-apoptotic activity [14].

DDR1 is widely expressed during embryonic development and in adult tissues. DDR1 knockout mice are smaller in size and exhibit defects in embryo implantation and mammary gland development [13], [16]. Upregulation of DDR1 in multiple human cancers including lung, breast, colon, esophagus, brain, ovary and prostate cancers implies that DDR1 is involved in tumor progression [2], [7], [8], [10], [14], [15], [16], [17]. Moreover, elevated expression of DDR1 in various fast growing invasive tumors indicates this RTK plays a critical role in cell adhesion, migration, invasion and subsequently metastasis [4]. Aside from its role in cancer, DDR1 has also been implicated in playing a key role in a variety of human diseases such as pulmonary fibrosis, pituitary adenoma, atherosclerosis, and congestive heart failure [14], [18].

In the present study, DDR1 gene silencing by siRNA or shRNA was used to elucidate the function of DDR1. Loss of DDR1 inhibited tumor cell proliferation, migration and invasion *in vitro* in numerous tumor cell lines. DDR1 silencing also impaired subcutaneous xenograft tumor growth in mice. In order to identify the downstream signaling of DDR1 kinase activation, we explored the effect of DDR1 downmodulation on the expression profile of the whole genome by microarray analysis. Here, we have identified *TGFBI* (transforming growth factor-beta induced) as one of several genes downstream of DDR1 signaling whose expression is modulated with DDR1 function.

*TGFBI* was originally identified in a human lung adenocarcinoma cell line as induced by transforming growth factor-beta (TGFβ) [19]. *TGFBI* is ubiquitously expressed in adult normal tissues, although downregulation of this gene has been found in various human tumor cell lines and in primary tumor specimens [20]. As an essential component of the ECM, TGFBI elicits numerous changes in cellular behavior, such as modification of cell adhesion and proliferation, inhibition of angiogenesis, deposition of extracellular matrix components and alteration of basement degrading enzyme products [20], [21]. TGFBI has a conflicting role in cancer progression. In some cases, overexpression of TGFBI in renal, pancreas or colon cancer cells induces cell migration and increases metastatic potential [22]. Others have shown that ectopic expression of *TGFBI* in transformed cells significantly suppresses tumorigenicity in multiple tumors, indicating that frequent downregulation of *TGFBI* is involved in tumor progression [21], [23]. Therefore, depending on the tissue, TGFBI functions as a promoter or suppressor of cancer growth [22].

We observed that loss of DDR1 induced *TGFBI* expression in a pancreatic tumor cell line at both mRNA and protein levels. Exogenous addition of TGFBI was able to mimic the knockdown effect of DDR1 *in vitro*. Taken together these data suggest that DDR1 may exert its oncogenic potential by downmodulating TGFBI levels, and TGFBI levels can be used as a marker associated with DDR1 downmodulation or inhibition.

## Results

### Generation of DDR1 knockdown in BXPC3 tumor cell model

**Figure 1:**
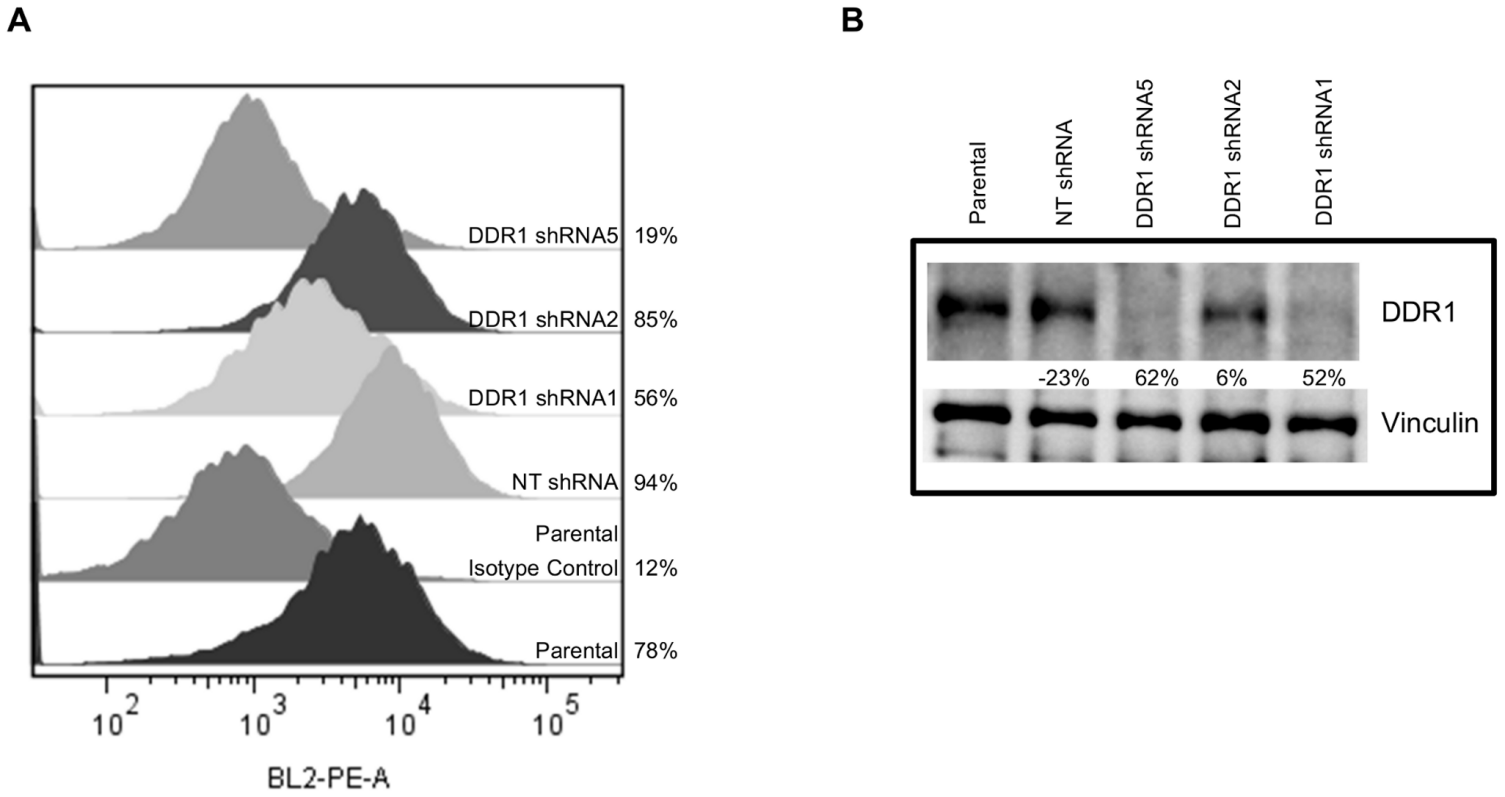
Generation of DDR1 knockdown in BXPC3 cells. BXPC3 cells were infected with the lentivirus transduction particles expressing three DDR1 shRNA sequences (1, 2 and 5) or a control non-target sequence (NT shRNA). At 48 hour post infection, the cells were selected with puromycin. DDR1 knockdown was confirmed (a) by flow cytometry and (b) by western blot analysis. Quantitation of flow cytometry was performed using FlowJo and data shown are percentages of DDR1 positive cells. Western data was analyzed with the Chemidoc software and the percentage of DDR1 knockdown was compared to the parental cells. Representative data of three independent experiments is presented.

To investigate the functional relevance of DDR1 in tumorigenesis, we generated stable DDR1 knockdown in BXPC3 via shRNA transduction. Initially, we evaluated three shRNAs against human DDR1 (shRNA1, 2 and 5) to silence DDR1 expression. Both shRNA1 and 5 showed the greatest suppression of DDR1 surface expression (Fig. 1a). Percentage of DDR1 positive cells was 78% in BXPC3 parental, 94% in NT shRNA, 56% in DDR1 shRNA1, 85% in DDR1 shRNA2, and 19% in DDR1 shRNA5 compared to the isotype control (12%). The expression of total protein was also validated by Western blot (Fig. 1b). Again, DDR1 shRNA5 showed the highest knockdown (62%) followed by DDR1 shRNA1 (52%) and shRNA2 (6%) cells compared to parental cells. NT shRNA did not show any decrease in DDR1 expression.

### DDR1 silencing suppresses BXPC3 clonogenic growth and migration *in vitro*

**Figure 2:**
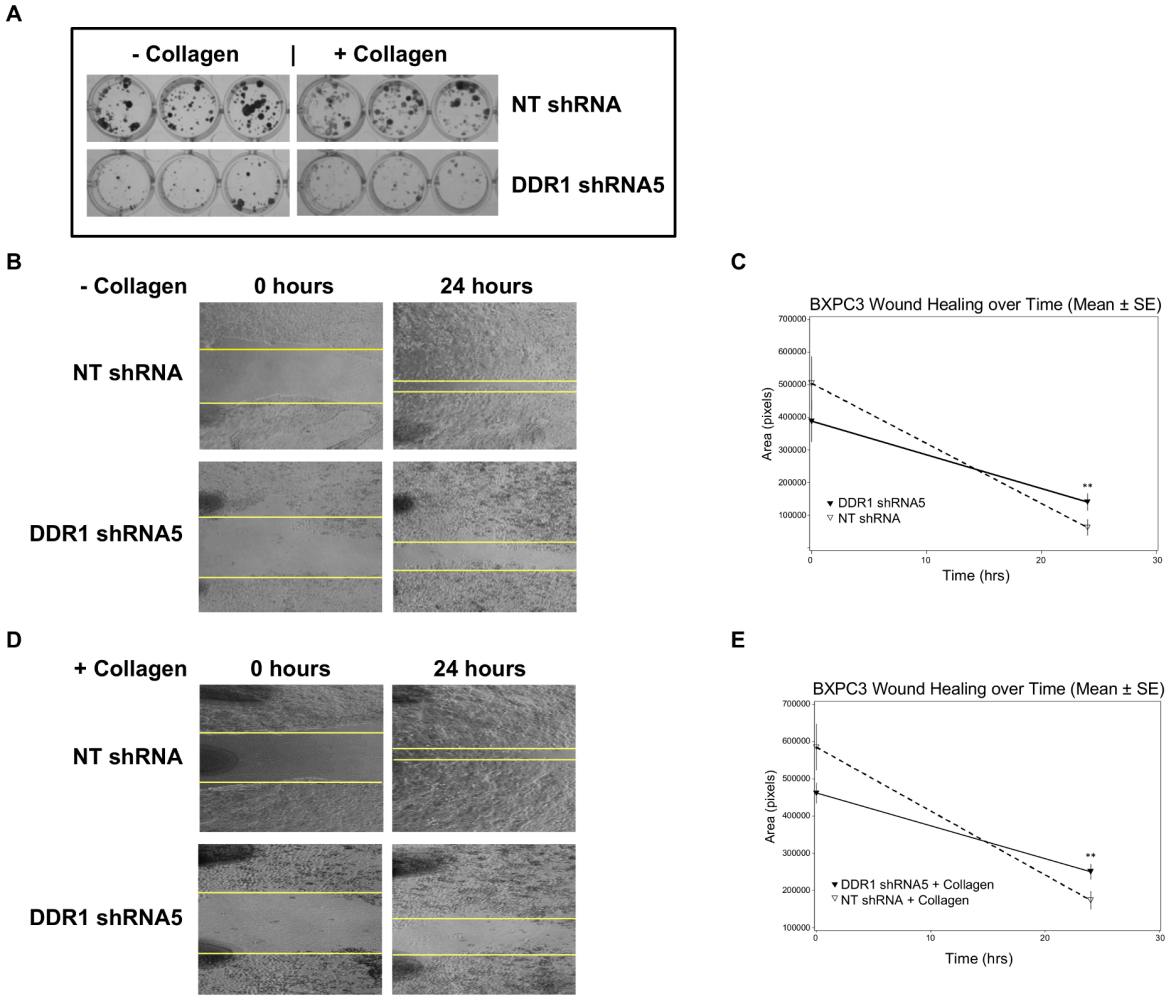
DDR1 knockdown inhibits BXPC3 clonogenic growth and migration. (a) BXPC3 cells transduced with either NT shRNA or DDR1 shRNA5 were compared in the clonogenic assay. The cells were seeded in 96 well plates with or without collagen and monitored for colony formation up to 10 days. The clones were visualized by crystal violet staining. (b) and (d) Effect of DDR1 knockdown was evaluated on BXPC3 cell migration with or without collagen using the wound healing assay. The wound closure at different time was photographed. (c) and (e) The rate of wound healing was analyzed by measuring the area with Image J program and plotted. NT shRNA (▽), DDR1 shRNA5 (▾) At study termination, p = 0.0004 (**) for both experiments.

To characterize DDR1 knockdown in functional assays, we first compared the growth of DDR1 shRNA5 and NT shRNA cells via colony forming assay with cells seeded on collagen-coated plates or tissue culture treated plates. We observed a decrease in the number and size of colonies with DDR1 shRNA5 cells by employing bright field images of crystal violet stained colonies (Fig. 2a).

To further assess the impact of DDR1 silencing on other cellular functions, we used the “wound healing” assay to measure cell motility *in vitro*. The wound area marked at “0 hours” is taken as 100% and the area closure at 24 hours was compared between the NT shRNA and DDR1 shRNA5 groups. In both the presence and absence of collagen, the control NT shRNA migrated faster than DDR1 shRNA5 and was able to close a larger wound area (Fig. 2b and 2d). The graphs show the calculated rate of wound healing over time (Fig. 2c and 2e). The calculated percent inhibition of “wound closure” by DDR1 shRNA5 was 36% (p = 0.0004) compared to NT shRNA without collagen. In the presence of collagen, the “wound closure” inhibited by DDR1 shRNA5 was 54% (p = 0.0004) compared to NT shRNA. The overall impact of DDR1 knockdown is statistically significant (p<0.05) by the Student's *t*-test. The data suggest that reduced expression of DDR1 significantly inhibited BXPC3 tumor cell migration, delaying wound closure. The phenotypes observed with NT shRNA cells was similar to parental BXPC3 cells when the assay was performed either with or without collagen (data not shown).

### DDR1 silencing inhibits BXPC3 tumor growth *in vivo*

**Figure 3:**
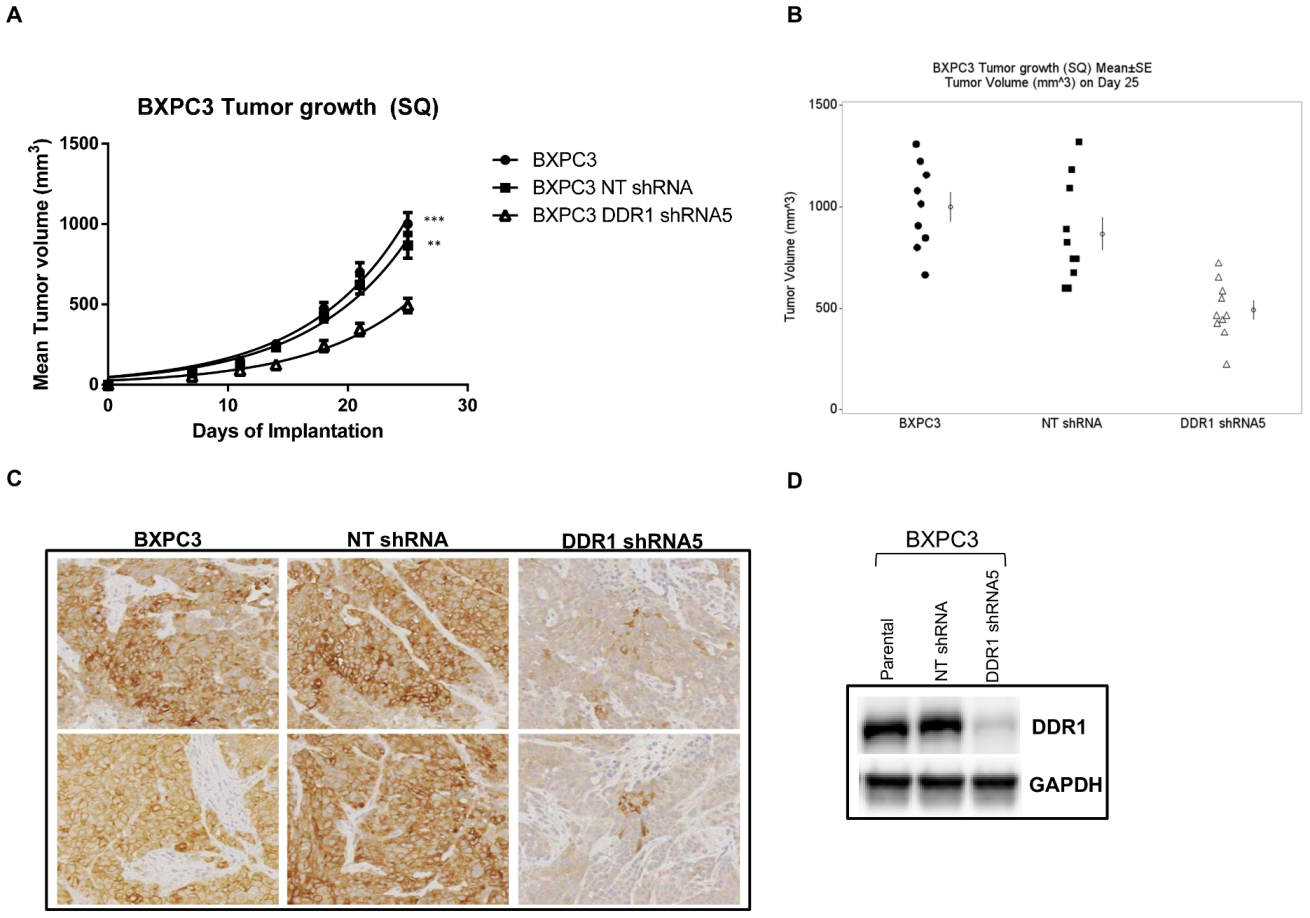
Efficacy of shRNA targeting DDR1 on BXPC3 tumor growth. (a) BXPC3 cells untreated (•), NT shRNA (▪) and DDR1 shRNA5 (Δ) were implanted in ICR-SCID mice and tumor growth was monitored up to 28 days. (b) On day 28, when compared to untreated and NT shRNA groups, the tumors in DDR1 shRNA5 group was the smallest as measured by the tumor volume (N = 10). The calculated percent inhibition in tumor growth in DDR1 shRNA group was 50.7% (***, p<0.0001) and 43.2% (**, p<0.0052) when compared to parental and NT shRNA groups, respectively. Tumors were harvested and DDR1 expression was confirmed by (c) immunohistochemistry and (d) western blot.

To evaluate whether DDR1 expression is required for tumor cell growth *in vivo*, we examined the effect of DDR1 knockdown on the growth of BXPC3 tumor xenografts in mice. Tumor growth monitored for 25 days showed that DDR1 knockdown resulted in approximate 50% tumor growth inhibition compared to the control groups (Fig. 3a). Statistical analysis of tumor size data on day 25 (Fig. 3b) indicated smaller tumors in the DDR1 shRNA5 group than those in the parental (p<0.0001) or NT shRNA group (p<0.01). To confirm DDR1 protein expression level *in vivo*, we evaluated DDR1 expression by immunohistochemistry and western blot in tumors at the end of the study. (Fig. 3c and 3d) Both analyses confirmed reduced DDR1 expression in the tumors from the shRNA5 group compared to the tumors from either the parental or the NT shRNA group. The loss of DDR1 expression in tumors might contribute towards attenuated tumor growth.

### Microarray analysis identifies pathway(s) modulated by DDR1 knockdown:

**Figure 4:**
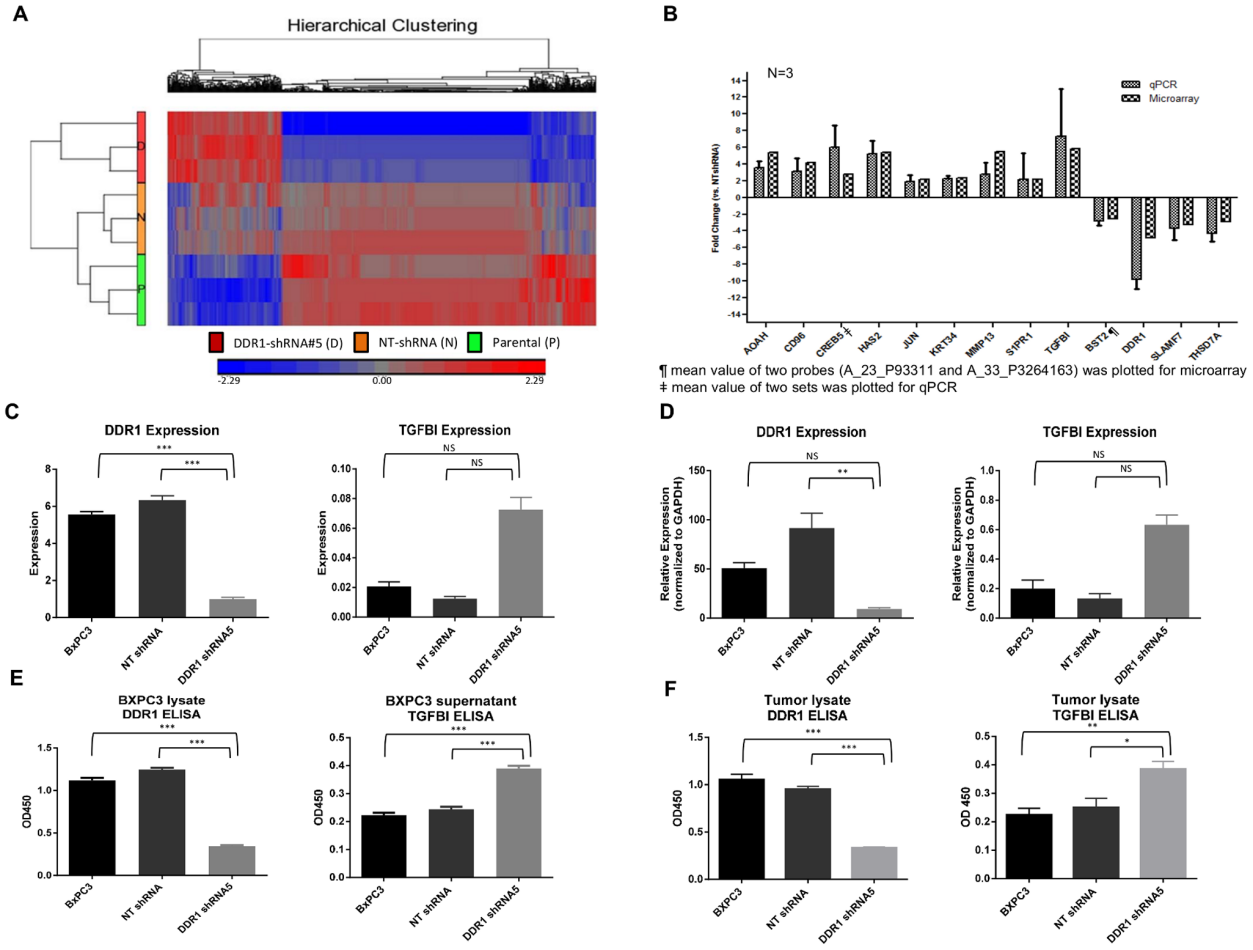
DDR1 knockdown induces upregulation of TGFBI. (a) Unsupervised hierarchical clustering of BXPC3 cells treated with DDR1 shRNA (D), NT shRNA (N) or untreated parental cells (P). (b) Expression validation of small subset of genes by microarray and qPCR. Mean value of two probes (A_23_P93311 and A_P3264163) was plotted for microarray. Mean value of two sets was plotted for qPCR. DDR1 and TGFBI expression in untreated, NT shRNA or DDR1 shRNA5 treated BXPC3 cells is measured by (c) microarray or (d) qPCR. (e) and (f) Relative DDR1 and TGFBI protein levels in untreated parental, NT shRNA or DDR1 shRNA5 treated BXPC3 cell lysates, supernatants or tumor lysates was measured by ELISA. Statistical significance is reported as *** (p<0.0001), ** (p≤0.005), * (p≤0.01), and NS (not significant).

To understand the biological role of DDR1 in tumorigenesis, we assessed the transcription profiles of BXPC3 parental, NT shRNA and DDR1 shRNA5 cells by microarray analysis. DDR1 shRNA5 cells clustered separately from untreated or NT shRNA cells, indicating an obvious effect of DDR1 knockdown on transcription profiles (Fig. 4a). One way ANOVA analysis employing a 2.0 fold cut off and p value <0.01 determined genes differentially expressed between DDR1 shRNA5 and NT shRNA groups. We identified a total of 1097 genes differentially expressed upon DDR1 knockdown and 586 genes in NT shRNA cells (Table 1), with 758 unique genes exclusively expressed by DDR1 knockdown (Fig. S1). Using this unique set of genes, GO analysis was performed to identify statistically enriched processes or pathways modulated upon DDR1 silencing. Gene ontology analysis by Partek generated a total of 49 categories (Fig. S2), with some categories relevant to different phenotypes observed *in vitro* like cell adhesion, wound healing and cell migration. Using the Broad_C2 gene lists for GO analysis, 149 terms were found to be statistically enriched. Using Ingenuity Pathway analysis (IPA) on the same set of genes Cellular Movement and Cellular Assembly and Organization were found to be among the top five networks (Fig. S3). Cellular Movement was the top molecular and cellular function which is complimentary to the migration and invasion phenotypes we observed in our models. Investigation of the subcategories related to wound healing showed that the size of lesion process is predicted to be downregulated based on the preponderance of overlapping genes being down regulated. Taken together, the GO analyses are concordant with phenotypic responses to DDR1 knockdown observed *in vitro* and *in vivo* such as reduced proliferation, invasion, migration, and wound healing.

**Table 1:** Significantly regulated probes in microarray studies (FC> = 2, p<0.01).

	DDR1 shRNA5 vs Parental	NT shRNA vs Parental	Unique to DDR1 shRNA5
**Up**	**520**	**437**	**230**
**Down**	**666**	**187**	**568**
**Total No. of Genes**	**1097**	**586**	**758**

### DDR1 knockdown modulates *TGFBI* expression

Microarray data was validated on a selected panel of genes by qPCR and was highly concordant between the two platforms (Fig. 4b). From both analyses, we identified *TGFBI* as one of the genes that was upregulated upon DDR1 knockdown in BXPC3 tumor cells. Downregulation of DDR1 expression induced *TGFBI* RNA expression by 4 fold (Fig. 4c and 4d). DDR1 knockdown in BXPC3 DDR1 shRNA5 was highly significant comparing with NT shRNA cells (p<0.001) in microarray and qPCR.

This observation was validated at the protein level using the TGFBI and DDR1 ELISA (Fig. 4e and 4f). In tumor cells and xenografts, DDR1 knockdown was highly significant compared to parental and NT shRNA cells (p<0.0001). Since TGFBI is a secreted protein, we checked TGFBI expression in both cell lysates and supernatants. A 2 fold increase in TGFBI expression was observed in DDR1 shRNA5 cells compared to parental and NT shRNA cells (p<0.0001). TGFBI level could only be measured in xenograft lysates, which again showed a similar increase in TGFBI expression in DDR1 shRNA5 xenografts (p = 0.0054 and 0.0134 compared to parental and NT shRNA xenografts, respectively). To our knowledge, this is the first report to link DDR1 and TGFBI expression.

### TGFBI mimics phenotypes observed upon DDR1 silencing in BXPC3 tumor cells

**Figure 5:**
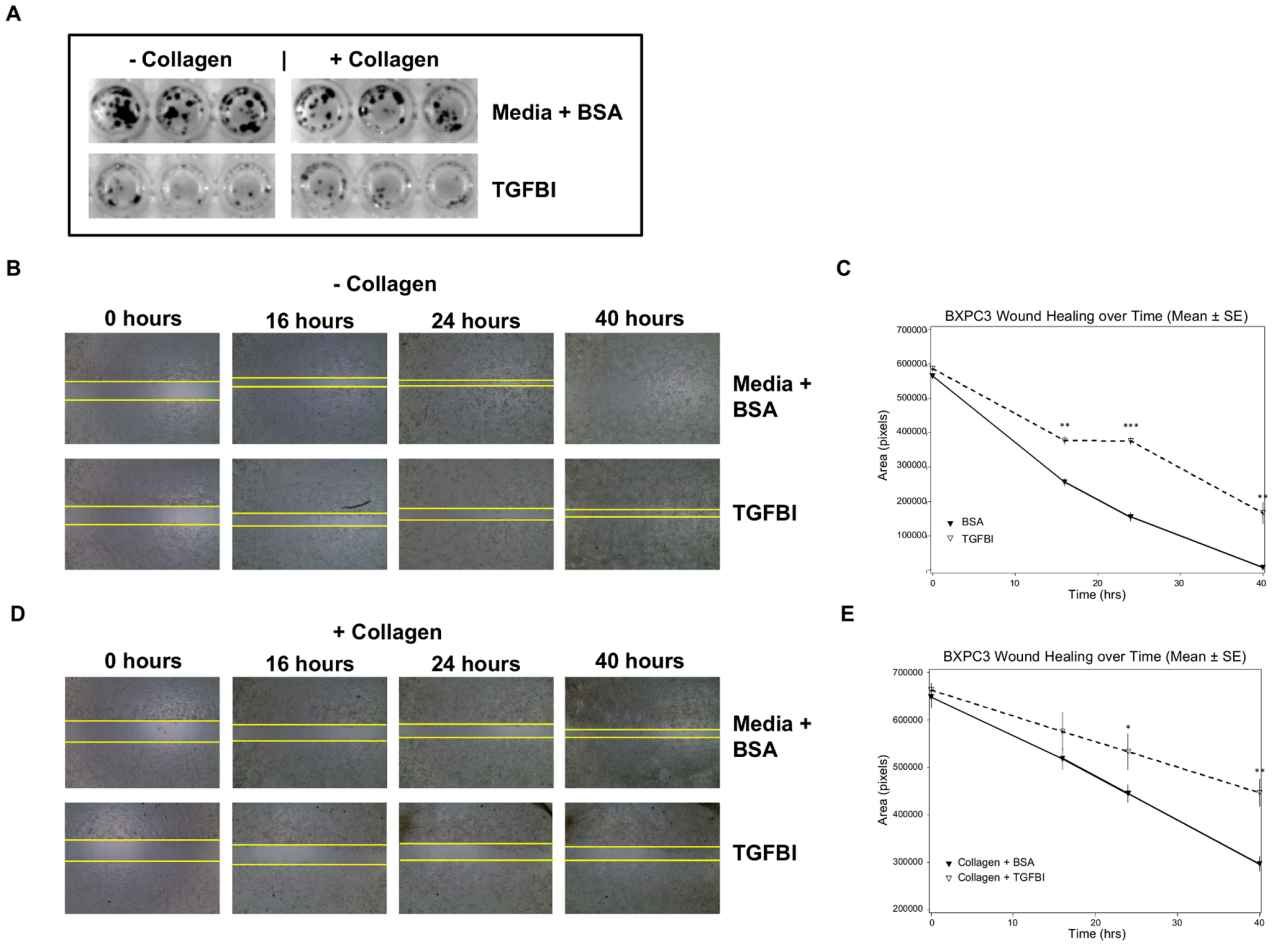
TGFBI modulates growth and migration of BXPC3 cells. (a) Exogenous addition of TGFBI completely eliminated BXPC3 colonies as determined by crystal violet staining. (b) and (d) Wound healing capacity of BXPC3 cells was reduced by TGFBI in the absence and presence of collagen. The area of wound closure was photographed at different time points over a period of 40 hours. (c) and (e) The rate of wound healing over time is presented here for both conditions. The area closure was measured using the Image J program. TGFBI treatment showed an overall 50% inhibition of wound healing (***, p<0.0001). In all cases media supplemented with BSA was used as a negative control. BSA (▾), TGFBI (▽).

We designed *in vitro* experiments to assess if exogenous TGFBI could recapitulate the phenotypes observed upon DDR1 silencing. BXPC3 cells were tested in both colony forming and wound healing assays in the presence of recombinant TGFBI with and without the addition of exogenous collagen. Based on a preliminary dose titration of TGFBI, we selected 50 µg/ml as the optimal concentration to use in functional assays that does not induce apoptosis (Fig. S4). Upon addition of recombinant TGFBI, we noticed very few colonies after 10 days both in the presence and absence of collagen (Fig. 5a). This robust growth inhibitory effect of TGFBI was similar to that observed upon DDR1 silencing. Fig. 5b to 5e show the effect of TGFBI on the wound healing process. At a longer incubation time of 40 hours, exogenous collagen shows modest inhibition of migration which is more pronounced in the presence of TGFBI. From the rate of wound healing calculated between 16 to 40 hours, recombinant TGFBI inhibited wound closure by 30%–50% compared to the control group (p = 0.0002). In the presence of collagen, TGFBI inhibited wound closure by 30–40% compared to the control collagen alone group (p = 0.0025). Under both conditions, TGFBI addition prevented wound closure at study termination.

### TGFBI can modulate the function of tumor models *in vitro*

**Figure 6:**
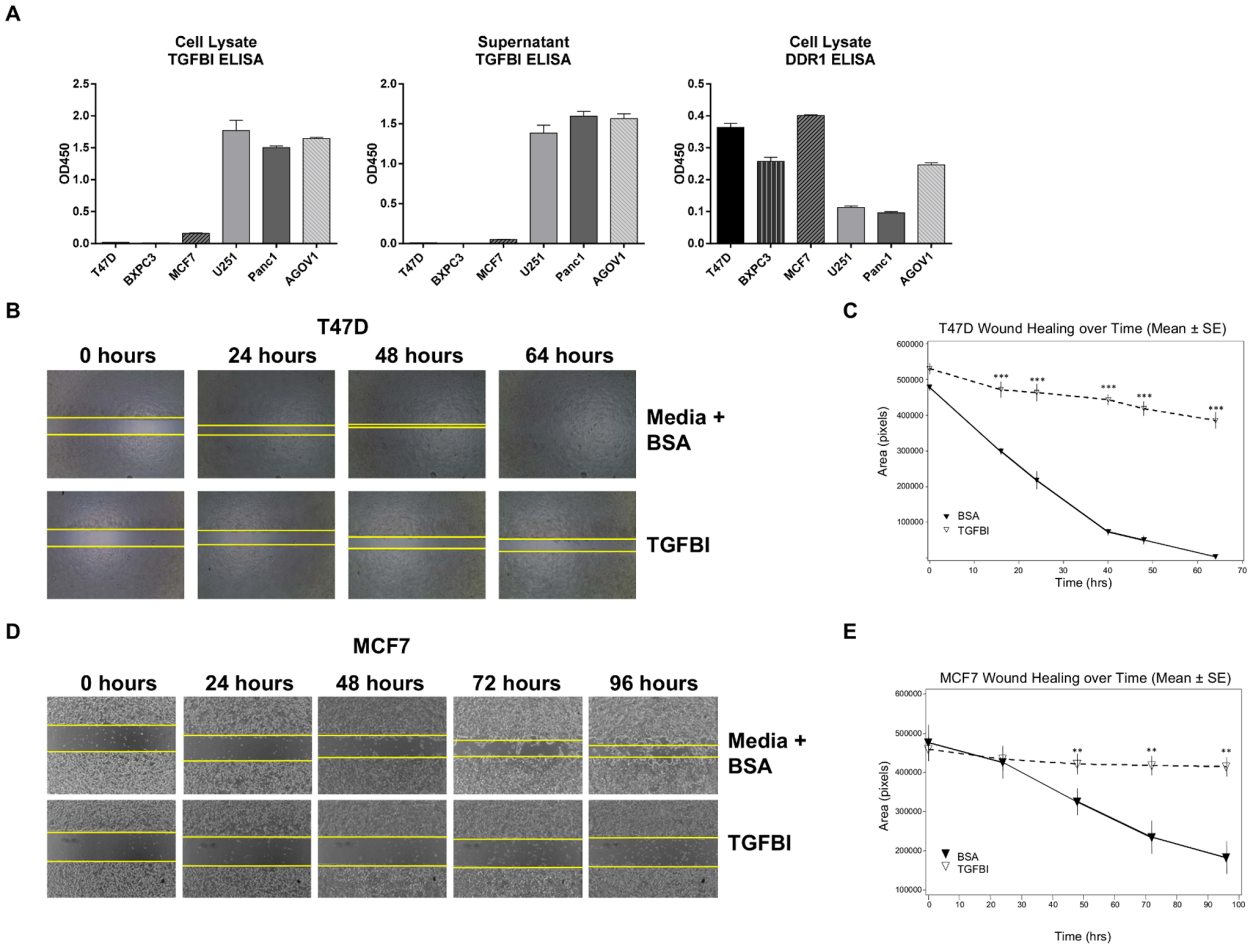
TGFBI inhibits lateral migration of T47D and MCF7 cells. (a) A small panel of tumor cells was tested for TGFBI and DDR1 expression via ELISA. TGFBI ELISA was run with both cell lysates and supernatants; DDR1 ELISA used only cell lysates. Exogenous addition of TGFBI completely abolished wound healing capability of T47D (b) and MCF7 (d) as earlier observed with BXPC3. The area of wound closure was photographed over time. The area closed at different time points was measured using the Image J program. The wound healing over time is plotted for T47D (c) and MCF7 (e). In all cases, media with BSA was used as a negative control. BSA (▾) TGFBI (▽) The data are representative of three independent experiments, each run in triplicate. Statistical significance is reported as *** (p<0.0001) and ** (p≤0.005).

Next, we screened a small panel of tumor cell lines to compare the levels of DDR1 and TGFBI expression by ELISA in order to identify additional models with similar expression profiles as BXPC3. We identified two breast carcinoma tumor cells, T47D and MCF7 (Fig. 6a), which demonstrated high DDR1 and low TGFBI, similar to BXPC3. We then evaluated these models in functional assays to see if a similar effect to BXPC3 could be achieved using recombinant TGFBI.

Both T47D and MCF7 cells lost their ability to migrate laterally upon TGFBI treatment. As shown in T47D (Fig. 6b and 6c), the percent inhibition in wound closure induced by recombinant TGFBI is 70–80% (p≤0.0001) calculated between 24 to 64 hours. In Fig. 6d and 6e, we observed a 48–85% inhibition in MCF7 cell motility (p = 0.009 at 48 hours, p = 0.0004 at 72 hours, p = 0.002 at 96 hours). Similar to BXPC3 cells, the wound could not be closed in either tumor cells in the presence of TGFBI at study termination.

We also examined the effect of recombinant TGFBI on T47D and MCF7 clonogenic growth. The effect was modest (data not shown). This could be due to the inherent nature of the cell types as neither of them grew well in single colonies compared to monolayer growth.

## Discussion

In this report, as a means to further elucidate the role of DDR1 in tumorigenesis, we present an important link between DDR1 and TGFBI. DDR1 silencing inhibited BXPC3 cell growth both *in vitro* and *in vivo* and modulated TGFBI expression. The unique finding from our study is the identification of TGFBI as a downstream effector molecule involved in the DDR1 pathway. Downregulation of DDR1 triggered induction of TGFBI expression. Therefore, we were interested to see if exogenous TGFBI could replicate the DDR1 knockdown phenotype *in vitro*. Recombinant TGFBI was capable of inhibiting tumor cell growth *in vitro* as well as wound healing in a similar manner although there was no change in DDR1 expression. We observed that DDR1 and TGFBI expression is reciprocal in several tumor cell lines (Fig. 6a). Further work would be required to understand the nature of DDR1-mediated effects on TGFBI expression and how downregulation of TGFBI contributes to tumorigenesis.

Cell migration plays a central role in processes like development, wound healing and cancer metastasis. The known functions of DDR1 include ECM remodeling, modulation of cell proliferation, promotion of cell adhesion and migration on collagen matrices [24]. Like other RTKs, DDR1 is also known to undergo proteolytic processing at the ectodomain by metalloproteases, which releases the ECD of membrane proteins and thus may regulate cell surface pool of DDR1. This shedding occurs both in a collagen dependent and independent manner, representing a major process of receptor regulation [25]. Overexpression of DDR1 in several tumor cell lines showed an enhanced transformed phenotype with increased anchorage independent growth and tumorigenic potential in nude mice [2]. DDR1 promotes metastasis through collective cell migration at the primary site [13] and has been identified as a critical component for bone metastasis in lung cancer [16]. Knockdown of DDR1 expression induced apoptosis in lung tumor cells and a dramatic reduction in bone metastasis, thus increasing survival rates in animal models of cancer [16]. Glioma cells with high DDR1 displayed enhanced migration and invasion, which could be reversed by an anti-DDR1 antibody [17]. Here, we report that silencing of DDR1 inhibited BXPC3 tumor cell growth and migration in vitro. This also led to reduced subcutaneous tumor growth *in vivo*. As mentioned earlier, DDR1 expression in heart and lung diseases may play a crucial role in regulating myocardial contractility, vascular remodeling, as well as in tissue microenvironment [14]. The fibroblast migration and proliferation with deposition of ECM protein is a major hallmark of fibrotic and wound healing processes. We believe that inhibition of lateral migration or wound healing observed as a result of lack of DDR1 expression provides a stronger argument towards the role of DDR1 in metastasis. A similar phenotype was observed by Miao's group where inhibition of DDR1 expression resulted in a decreased migration and invasion of NSCLC cells [4]. Reports from various laboratories including our current work have shown a strikingly high level of DDR1 expression in a number of fast growing invasive tumors leading to metastasis, which suggests the involvement of this RTK in proliferation and stromal invasion of tumors [2], [26]. This again supports the hypothesis that interactions of DDR1 with ECM play a role in cell growth, adhesion, cell-cell interaction, migration and invasion or metastasis.

Collagen-interaction is potentially important for controlling cell shape and movement via interaction between two Collagen receptors, the integrins and DDR1, although they employ fundamentally different collagen binding modes. While integrins are the primary modulators of cell adhesion and migration, DDR1 can also influence these processes in a cell-type dependent manner. Little is known about the interplay between the DDRs and collagen binding integrins, but a cross-talk between them was first identified by Shintani [27]. His group showed that collagen-induced BXPC3 cell scattering was dependent on the coordinated activities between integrin β1 and DDR1; this in turn activated the JNK pathway via the p130 cas scaffold and caused an upregulation of N-Cadherin expression. This complex interaction involved both collagen receptors along with their effector molecules, FAK and PYK2 and the downstream signaling pathways from both converged. Here, knockdown of both integrin β1 and DDR1 was necessary for complete inhibition of collagen-induced cell scattering. The cooperation of DDR1 and α2β1 helped to mediate the collagen-induced epithelial-mesenchymal transition. DDR1 is also known to counteract the signaling effects of the α2β1 integrin by reducing the activation of STAT1/3 and Cdc42 [25]. Likewise, Yeh, et al. reported that DDR1 inhibited collagen induced cell extension by suppressing α2β1 integrin which normally activates Cdc42; hence, DDR1 activation caused increased junctional stability [13]. These opposing functions of DDR1 and α2β1 might regulate many cellular events and the signaling balance between these two pathways, can be critical in determining cell fates. DDR1 activation upon Collagen-I stimulation was confirmed in both BXPC3 and T47D. Although exogenous collagen has a modest effect on clonogenic growth and lateral migration of tumor cells, the DDR1 knockdown phenotype remains the same either in the presence or absence of collagen. The observed functional effect of DDR1 on cell growth and migration independent of exogenous collagen supplementation could possibly be explained by endogenous expression of collagen providing constitutive low level DDR1 activation. Alternatively, direct modulation of DDR1 signaling can be achieved solely by the high level of DDR1 expression in pancreatic and breast cancer models.

The *TGFBI* gene which is mapped to chromosome 5q31 is known to be deleted in several human cancers including renal, lung, esophageal cancers and leukemias. Downregulation of TGFBI is reported in tumor cell lines and in patients with lung and breast tumors [20]. *In vitro* studies have shown an association of TGFBI in maintaining microtubule stability and inhibiting tumorigenicity and tumor angiogenesis [20], [28]. The TGFBI null mice, were reported to be prone to spontaneous tumor formation and showed more metastatic potential than TGFBI+/+ mice [22]. However, an acquired expression of TGFBI by SW480 colon cancer cells leads to a more aggressive phenotype of metastasis by favoring extravasation [20]. Hence, depending on tumor type, TGFBI may show dual activities, either as a tumor promoter or a tumor suppressor and contribute to tumorigenesis accordingly.

TGFBI is involved in similar cellular functions as DDR1 like cell proliferation, migration and cell matrix interaction [29]. As a secreted protein, TGFBI, is primarily expressed in collagen rich tissues along with other ECMs. The RGD sequence in the C-terminal region of TGFBI is thought to act as a universal ligand recognition site for integrins, another collagen receptor [20] and thus, TGFBI participates in cellular processes via interaction with integrins where signaling through FAK and Rho-A can be engaged [28], [30]. Shelton and Rada also showed that TGFBI inhibited the attachment of human scleral fibroblasts (HSFs) to collagen I, and this might limit cell matrix interaction between HSFs and Collagen-I via integrin receptors [31]. All these observations indicate that the interaction between integrins and TGFBI is well validated, which in turn may modulate the role of TGFBI in cellular functions.

In the present study, we documented an association between DDR1 and TGFBI for the first time. We demonstrated that silencing of DDR1 results in an upregulation of TGFBI that could block tumor cell growth and motility, while exogenous addition of TGFBI into tumor cells recapitulated those phenotypes. Microarray profiling of cells lacking DDR1 identified several additional genes which were modulated upon DDR1 silencing; some of those had been previously reported to be involved in different processes of tumorigenesis, cell motility as well as ECM remodeling. Our small panel of gene list includes c-jun, MMP-13, CREB, and HAS2. DDR1 signaling via JNK1 pathway involves c-jun activation [27]. Hyaluronan synthetase, HAS2 overexpression and amplification have been implicated in tumor proliferation and metastasis in genitourinary tumors. DDR1 and DDR2 both play significant roles in the expression of several MMPs [18]. Overexpression of MMP-13 in several types of malignancy increases the invasive capacities of the malignant cells and has been linked with shorter overall patient survival [32]. MMP-13 and HAS2 are also constituents of the ECM and participate in wound healing, ECM modeling, and tissue repair [33], [34], [35], [36]. Upregulation of HAS2 and MMP-13, in a similar fashion as TGFBI may imply their collective role in tumor progression. Interestingly, TGFBI was found to be upregulated in MCF-7 cells overexpressing c-jun [37]. Again, MMP-13 and TGFBI expression is elevated in esophageal squamous cell carcinoma [38]. In a study with TGFBI null mice, loss of TGFBI promoted cell proliferation via aberrant activation of the CREB-cyclin D1 pathway and predisposed mice to spontaneous tumor development [20]. Identifying these genes along with TGFBI via microarray while characterizing DDR1 knockdown cells provides stronger validation to our finding and analysis.

Finally, our study provides a functional connection between DDR1 and TGFBI. We demonstrated that silencing of DDR1 inhibited tumor cell growth and motility, and induced TGFBI expression, both *in vitro* and *in vivo*. Our hypothesis indicates this tumorigenic effect of DDR1 appears to be in part mediated via TGFBI downregulation, which reinforces the notion of a tumor suppressor role for TGFBI We have also demonstrated that exogenous TGFBI could recapitulate the phenotypes associated with DDR1 knockdown. In conclusion, our study not only adds more clarity to how DDR1 may be mediating tumorigenesis, but also supports the tumor suppressor role of TGFBI and suggests that TGFBI may serve as a biomarker for modulators of DDR1. Our observation points towards the value of both DDR1 and TGFBI in tumorigenesis. Further investigation would be needed to understand the interaction between TGFBI and DDR1, which in turn may favor developing therapeutics for either or both.

## Materials and Methods

### Ethics statement

All studies conducted in laboratory animals described in this manuscript were carried out in strict accordance with the applicable sections of the Guide for the Care and Use of Laboratory Animals from the National Research Council. The protocol and any amendments or procedures involving the care or use of animals in these studies were reviewed and approved by Agensys or Charles River Institutional Animal Care and Use Committee before the initiation of such procedures. All efforts were made to minimize pain and suffering. If an animal was determined to be in overt pain/distress, or appeared moribund and was beyond the point where recovery appeared reasonable, the animal was euthanized for humane reasons in accordance with the AVMA Guidelines on Euthanasia.

### Cell Culture

BXPC3 (Human pancreatic adenocarcinoma), T47D (Human ductal breast epithelial tumor cell) MCF7 (Human breast adenocarcinoma), Panc1 (Human pancreatic epithelial carcinoma) cells were obtained from ATCC (Manassas, VA). U251 (Human glioblastoma astrocytoma) cell was part of NCI-60 tumor cell panel. The AGOV1 tumor cell has been established in-house from a patient tumor (Human ovarian adenosquamous carcinoma). All tumor cells were and maintained in appropriate growth media containing 10% (v/v) fetal bovine serum (Omega Scientific) and 2 mmol/L glutamine. Non targeting (NT) short hairpin (SHC002V; Mission, Sigma, St Louis, MO) and specific shRNAs for human DDR1 were used to derive knockdown cells. The shRNA sequences are listed in Table 2.

**Table 2:** DDR1 shRNA Sequence.

**1**	**TRCN0000010084**	**CDS**	**CCGGGTGTGGCTCGCTTTCTGCAGTCTCGAGACTGCAGAAAGCGAGCCACACTTTTT**
**2**	**TRCN0000010085**	**CDS**	**CCGGGGACTATATGGAGCCTGAGAACTCGAGTTCTCAGGCTCCATATAGTCCTTTTT**
**5**	**TRCN0000121082**	**3UTR**	**CCGGCCTATACGTTTCTGTGGAGTACTCGAGTACTCCACAGAAACGTATAGGTTTTTG**

### Colony Formation Assay

For colony formation, cells (1.5×10^3^/well) were seeded into 96 well tissue-culture treated plates (BD Falcon) and colony growth was measured by crystal violet (Sigma, St Louis, MO) staining after ten days, and then photographed. Fresh recombinant TGFBI (R&D systems) (50 µg/ml) or BSA (5%) was added at every 72 hours, in appropriate experiments. If appropriate, Collagen-I (BD Biosciences) was either added during the start of the experiment or used to coat the plates at 10 µg/ml. Each experiment consisted of three replicates and was repeated three times.

### *In vitro* “wound healing” assay

The Ibidi 24 well culture inserts (Ibidi, Verona, WI) were used to monitor wound healing or cell motility. Based on the growth pattern, 1–10×10^4^ tumor cells were seeded per well in a 24 well plate. Once the cells reached confluence, inserts were removed, washed, and fresh medium was added to the wells. TGFBI (50 µg/ml) or BSA (5%) was added after removal of inserts, as appropriate. Collagen-I (BD Biosciences) was added at 10 µg/ml as needed. Images were captured at given time points using the EVOS microscope (Advanced Microscopy Group). Migrated area was calculated using Image J (NIH software package) to determine the rate of migration. The data were plotted as percent area closure over time. Each experiment was performed three times in triplicate and Student's *t*-test was employed for statistical analysis.

### Flow cytometry

Cell surface expression of DDR1 was confirmed via flow cytometry using a phycoerythrin conjugated mouse anti-human DDR1 monoclonal antibody (Biolegend) or a mouse IgG3 isotype control (Biolegend), at a final concentration of 5 µl/test at 4°C for 1 h. The fluorescence intensity was detected using the Attune flow cytometer (Life technologies, Carlsbad, California); the data were analyzed with FlowJo v7.6.5 (Tree Star).

### Western blotting

Cells were lysed in RIPA buffer (Thermo Scientific, Pittsburg, PA) with complete protease and phosphatase inhibitor cocktails (EMD Millipore, Billerica, MA) and equal proteins were separated on 4–12% Tris-Glycine gel (Biorad Laboratories) under reducing conditions. Proteins were transferred to nitrocellulose membranes, and probed with primary antibodies and appropriate secondary HRP-conjugated antibodies. Immunoreactive proteins were visualized by an enhanced chemiluminescence reagent (Thermo Scientific) and photographed in the Chemidoc (Biorad Laboratories). The antibody sources are: anti-DDR1 (Santa Cruz Biotechnology, Dallas, TX), anti-Vinculin (Millipore, Billerica, MA), and anti-GAPDH (Santa Cruz Biotechnology, Dallas, TX). All secondary antibodies were purchased from Jackson Immunoresearch Lab.

### Xenograft tumor growth

BXPC3 tumor cells (2×10^6^ per mouse) either expressing NT (non-target) shRNA or DDR1 shRNA5 or the parental cells were injected subcutaneously into the flank of each ICR-SCID mouse (Taconic Farms, Germantown, NY). Tumor growth was monitored by caliper twice a week until study termination. Tumor volumes were analyzed by the Kruskal-Wallis test and pairwise comparisons were made using Tukey's test procedures (2-sided) on the ranked data to protect the experiment-wise error rate.

### Immunohistochemistry

The xenograft samples collected at the end of study were processed for immunohistochemistry. Formalin-fixed, paraffin-embedded (FFPE) tumor tissue sections were stained for DDR1 by using a mouse anti-human DDR1 monoclonal antibody (developed in-house). The antibody was validated to specifically stain DDR1 in human tumor tissues. Appropriate positive and negative controls were included in the staining process. After staining, the slides were evaluated under the Nikon Eclipse E400 microscope equipped with SPOT digital camera and images were captured.

### ELISA

We developed an ELISA to measure DDR1 and TGFBI expression levels in tumor cells, conditioned media and xenograft samples. For the DDR1 ELISA, mouse anti-human DDR1 monoclonal antibody mixture was used for capture. For total DDR1 expression, a rabbit anti-human DDR1 (C-20) antibody (Santa Cruz) and anti-rabbit HRP (Southern Biotech) were used for detection. For the TGFBI ELISA, an anti-BIGH3 (TGFBI) monoclonal antibody (R&D Systems) was used for capture and a biotinylated anti-BIGH3 (R&D Systems) antibody and Streptavidin-HRP (Southern Biotech) were used for detection. All lysates were quantitated by BCA to ensure equal loading. Data are reported as OD values to compare relative levels of TGFBI and DDR1. All measurements were performed in triplicate and the Student's *t*-test was used for statistical analysis.

### RNA preparation and real time PCR

Total RNA was isolated using TRIzol Reagent (Life Technologies, Carlsbad, USA) and RNA quality and quantification were analyzed by RNA Nano 6000 chips in a Bioanalyzer 2100 (Agilent Technologies, Santa Clara, USA). cDNA was synthesized with Superscript III RT (Life Technologies). The primers used for qPCR were designed in Primer3 (See Fig. S5 for primer sequences). Reactions were performed in triplicate on a CFX384 Real-Time PCR machine (Biorad Laboratories). Ct values and melting curves were determined in CFX Manager Version 1.5 (Biorad Laboratories). Relative gene expression was calculated by normalization to GAPDH using the 2∧-ddCt method [2].

### Microarray whole genome expression profiling

All treatment groups were run in triplicate as biological replicates. Cyanine 3-CTP labeled cRNA target was prepared using One-Color Low Input Quick Amp kit and One Color Spike-In kit (Agilent Technologies, Santa Clara, USA) using 200 ng input RNA (RIN>8.0). Specific activity of Cyanine 3 was calculated in pmol/µg with a minimum threshold of 15 pmol/µg. 1650 ng of labeled cRNA was fragmented and hybridized onto Agilent Human GE 4×44 K v2 microarrays. Hybridization was done for 17 hours at 65°C by rotating at 10 rpm. Microarray slides were scanned in an Agilent Scanner using profile AgilentHD_GX_1Color at Green PMT 100%. The scanned image was extracted to data using Agilent Feature Extraction Software version 10.

### Microarray Datasets and Analysis

All data described in this manuscript are MIAME compliant and have been deposited in NCBI's Gene Expression Omnibus Database (http:www.ncbi.nlm.nih.gov/geo/) under GEO Series accession number (GSE39207). Data analysis was performed and gene lists were generated in Partek Genomics Suite v6.6 (Partek, St. Louis, USA). GO enrichment analysis was performed to C2: curated gene sets of MSigDB v3.8, which had more than 50 genes in the gene set, applying Fisher's exact test. Networks and pathways were investigated with Ingenuity Pathway Analysis software (IPA) (Ingenuity Systems, Mountain View, CA, USA; http://www.ingenuity.com).

## Supporting Information

Figure S1 **Genes regulated in DDR1 shRNA5 after excluding NT shRNA.** Differentially expressed genes unique to knockdown by DDR1 shRNA5 as determined by one way ANOVA, FC≥2.0, p value <0.01.
(XLSX)

Figure S2 **Gene ontology analysis using differentially expressed gene list unique to knockdown by DDR1 shRNA5.** GO: Gene ontology analysis performed with Partek software. GO terms with DDR1 or TGFBI: GO categories identified by Partek software containing DDR1 or TGFBI. BROAD_C2: GO analysis using Broad_C2 curated gene sets. BROAD_C2 DDR1 or TGFBI: Statistically enriched Broad_C2 gene sets containing DDR1 or TGFBI. BROAD_C2 gene lists: Genes contained within highlighted Broad_C2 gene sets.
(XLSX)

Figure S3 **IPA Analysis-Wound Healing.** Results of Ingenuity Pathway Analysis using differentially expressed gene list unique to knockdown by DDR1 shRNA5. Predicted impacts on selected functions based on gene expression changes within genes in dataset.
(XLSX)

Figure S4 **TGFBI does not induce apoptosis in BXPC3 cells.** BXPC3 cells (1×10^4^/well) were seeded into 96 well tissue-culture treated plates (BD Falcon). After 24 hours, recombinant TGFBI (R&D systems) or BSA (5%) was added at 100, 50, 25, and 12.5 µg/ml. Cell viability was determined 48 hours later using Presto Blue (Life Technologies). Apoptosis was measured by Caspase 3/7 activity using the Caspase-Glo 3/7 Assay (Promega). Both fluorescence and luminescence was measured using the Synergy H4 instrument (Biotek). Data were analyzed and reported by normalizing the apoptosis activity to total cell number and comparing the fold-change of TGFBI-treated cells to the untreated group. The experiment consisted of three replicates per treatment.
(TIF)

Figure S5 **qPCR primers for microarray validation.**
(DOCX)
