# Supplementary figures and images for: Discoidin Domain Receptor 1 Contributes to Tumorigenesis through Modulation of TGFBI Expression
Source: PLoS One. 2014 Nov 4;9(11):e111515. doi: 10.1371/journal.pone.0111515 (PMC4219757; doi:10.1371/journal.pone.0111515)

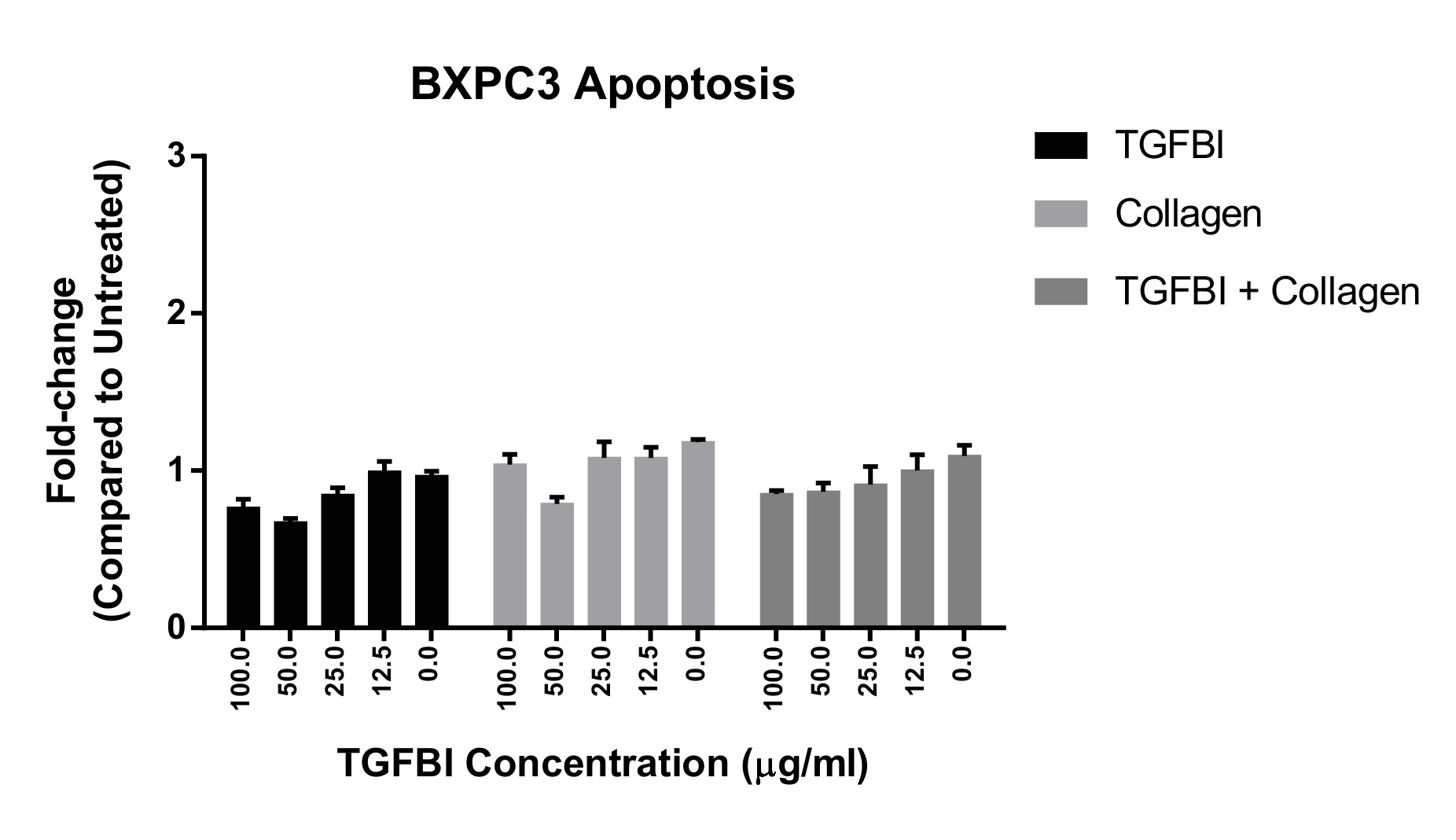

Supplement: Figure S4 — TGFBI does not induce apoptosis in BXPC3 cells. BXPC3 cells (1×10^4^/well) were seeded into 96 well tissue-culture treated plates (BD Falcon). After 24 hours, recombinant TGFBI (R&D systems) or BSA (5%) was added at 100, 50, 25, and 12.5 µg/ml. Cell viability was determined 48 hours later using Presto Blue (Life Technologies). Apoptosis was measured by Caspase 3/7 activity using the Caspase-Glo 3/7 Assay (Promega). Both fluorescence and luminescence was measured using the Synergy H4 instrument (Biotek). Data were analyzed and reported by normalizing the apoptosis activity to total cell number and comparing the fold-change of TGFBI-treated cells to the untreated group. The experiment consisted of three replicates per treatment. (TIF) [file pone.0111515.s004.tif]
