# Supplementary material for: Discoidin Domain Receptor 1 Contributes to Tumorigenesis through Modulation of TGFBI Expression
Source: PLoS One. 2014 Nov 4;9(11):e111515. doi: 10.1371/journal.pone.0111515 (PMC4219757; doi:10.1371/journal.pone.0111515)
Supplement: Figure S5 — qPCR primers for microarray validation. (DOCX) [file pone.0111515.s005.docx]

AOAH: (F) GTAATGGCATTTGGGGTGTC, (R) TCTCCCAGCAAAATGATTCC

CD96: (F) AACCAGCCCAATCAGACAAC, (R) GTGGAGGGTCTGTTGAGGAA

CREB5: (F) TTACAATAGAATAATTTAGCCTCAGGA, (R) AAAGGAGAAAACTAGAATCTAATGAGC

HAS2: (F) GGGGGAGATGTCCAGATTTT, (R) ATGCACTGAACACACCCAAA

JUN: (F) TAACAGTGGGTGCCAACTCA, (R) TTTTTCTCTCCGTCGCAACT

KRT34: (F) TTTCCTGACCCAAGCAAAGA, (R) AAACAGGCTCGACCCTCAAC

MMP13: (F) TGGTCCAGGAGATGAAGACC, (R) TCCTCGGAGACTGGTAATGG

S1PR1: (F) AACTGACCTCGGTGGTGTTC, (R) TACATGGGTCGGTGGAATTT

TGFBI: (F) GGGAAGTCCTGGCACAGTT, (R) CCTCCAAGCCACGTGTAGAT

BST2: (F) TGCTGGGGATAGGAATTCTG, (R) CATTGCGACACTCCATCACT

DDR1: (F) CCCTTCCTGTTCTTCCTTCC, (R) GGGCCATGTCCAGTGTCTAT

SLAMF7: (F) GAAGCAGATGCACCTGACAA, (R) ATTTCATGAGGGCAGACAGC

THSD7A: (F) AGGCGCTTAACCAGTATTGC, (R) GCTGCCATCCAAACTGAACT

GAPDH: (F) AGAACATCATCCCTGCCTCTACTG, (R) AAATGAGCTTGACAAAGTGGTCGT
